# Complications during the Management of Dislocated Tracheostomy Cannula

**DOI:** 10.1155/2019/5346262

**Published:** 2019-05-13

**Authors:** Kristian Krogh, Anette Marianne Fedder

**Affiliations:** ^1^Department of Intensive Care Medicine, Aarhus University Hospital, Palle Juul-Jensens Boulevard 99, 8200 Aarhus N, Denmark; ^2^Research Center for Emergency Medicine, Aarhus University Hospital, Palle Juul-Jensens Boulevard 99, 8200 Aarhus N, Denmark

## Abstract

We describe an acute complication to a tracheostomy cannula in the form of a dislocated cannula after a surgical tracheostomy in a 65-year-old male patient. The case illustrates the development of progressing subcutaneous emphysema resulting in a cannot intubate, cannot oxygenate (CICO) situation and the airway management of the patient with respiratory distress. Early recognition and situational awareness are essential in the management of patients with acute airway complications. Consequently, deliberate practice and continuing professional development should be encouraged so that we can best manage acute situations when they occur.

## 1. Introduction

While tracheostomy cannulations are commonly used in intensive care units (ICUs) and the procedures around cannula insertion, maintenance, and decannulation are well described [1, 2], there are several complications related to all procedures related to cannula use [3, 4]. Some of these are trivial where others are life-threatening. Early recognition of these potential severe complications is essential for timely intervention. Pattern recognition and intervention are possible due to situational awareness [5], something that is promoted through practice and by reflection-on-action [6, 7].

## 2. Case Presentation

The patient, a 65-year-old male, was admitted to ICU after thoracic surgery, with a mitral valve replacement and a CABG (coronary artery bypass graft), in 2017. The patient, who had a kidney transplant in 2010, developed postoperative kidney failure and continuous renal replacement therapy (CRRT) dialysis was commenced. Furthermore, the patient developed atrial fibrillation, pulmonary oedema, and subsequently respiratory distress. Thus, the patient was orally intubated, as intermittent CPAP (Continuous Positive Airway Pressure) or periods with NIV (noninvasive ventilation) were insufficient to maintain sufficient respiratory support. Attempts to extubate were unsuccessful, and, on the sixth postoperative day, it was decided to perform a surgical tracheostomy, which was made with a tracheostomy tube size 8 inserted. Over the next days, patient's respiratory status was improving, and the patient was at times able to maintain saturation (SaO_2_) at 95-98% on 3-5L/min 100% oxygen on a speaking valve up till 7 hours per day. During this, the patient was developing delirium and was fingering his tracheostomy tube, even with increasing effort to attenuate his symptoms.

Before the incident, there had been two reinsertions of the tracheostomy tube that had both proven problematic but possible, as such if the problem was persisting, the patient could be intubated orally (previous Cormack Lehane grade (CL) 1), which was the information given at handover at the beginning of the night shift.

When called to assist the patient at approximately 11 pm, there had been one failed attempt to reinsert the tube by the nurse. The patient was wheezing but managing on speaking valve with a SaO2 above 96%. First attempt to reinsert the tube was unsuccessful. Following this, was the use of a suction catheter as a guide attempted, after removal of the speaking valve with a flow of 5L oxygen, but unsuccessful, however.

The patient expresses increased difficulty, breathing became more restless, and the patient attempted to grab the tube. Therefore, the speaking valve with oxygen flow was reconnected, as a flow of 5L had been sufficient to maintain SaO_2_ prior to the attempted replacement of the tube.

From this point, subcutaneous emphysema started to develop almost immediately, and the speaking valve was disconnected right away. As soon as a ventilation bag/mask was ready, the tracheostomy tube was removed, and a nurse covered the tracheostomy while the patient was preoxygenated, and preparations for a rapid sequence induction for oral intubation of the patient were made, having the difficult airway trolley and video laryngoscope (a Storz C-MAC video laryngoscope) readily accessible by the bed.

As soon as equipment and drugs were at ready at hand, the rapid sequence induction was made, and the patient was attempted intubated with a size 8 tube with a guide using a Mac 3 blade on a standard laryngoscope (CL 2). Nevertheless, the tube met resistance and could not be inserted. The second attempt was made, using video laryngoscope, vocal cords fully visible, size 8 tube inserted but could not be advanced into the trachea. Call for help from another anaesthetist was made and a third attempt was made using a size 7 tube, same problem. Saturation dropped below 80, and the patient was bag/mask ventilated. When help arrived, the patient's saturation had been elevated to 94-96 percent as ventilation was still possible, although subcutaneous emphysema was progressing. Meanwhile, a nurse had been asked to retrieve and ready a flexible bronchoscope.

**Figure 1:**
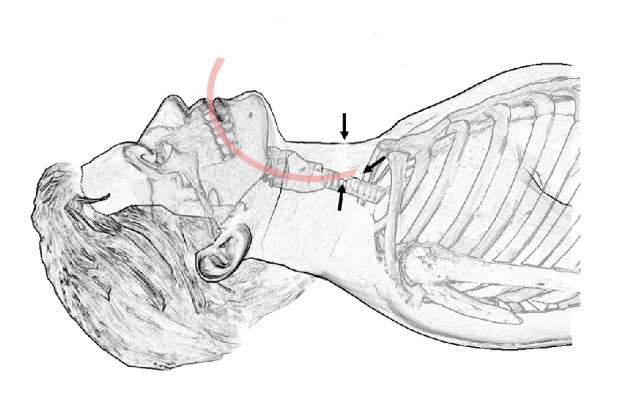
Visual presentation of the tube and the via falsa anterior to the trachea (arrows pointing to the tracheostomy and the via falsa).

The second anaesthetist toke over and attempted intubation with a size 6 tube. Vocal cords were fully visible, but the progression of emphysema had limited the view since the first attempt was made. The tube was inserted to 24 cm mark using some force, the cuff was inflated, and ventilation was attempted. There was no sound when auscultation was made, no CO_2_ returned, and saturations started to fall. Hereafter, bag-mask ventilation was no longer possible and a cannot intubate, cannot oxygenate (CICO) situation was present, and saturation plunged further. Size 6 tube was reinserted, past vocal cords that were just visible due to the still increasing intratracheal emphysema. It was not possible to ventilate the patient. Meanwhile, equipment for cricothyrotomy (a tracheal hook and scalpel for rapid four-step technique) had been requested. Palpation was at this point extremely difficult due to the massive subcutaneous emphysema, and digital exploration of the surgical tracheostomy was performed. The tracheal cartilage cranial from incision was palpable, and the endotracheal tube was found exciting the trachea into a via falsa anterior to the trachea as shown in Figure 1. Subsequently, the tube was pulled back at digitally guided into the trachea, where the cuff was inflated, and tube could be held in place supporting the inflated cuff with a finger. Ventilation was now possible, and saturation started to rise and reached 95% shortly after that.

Following that the thoracic surgeon called (no in-house ENT surgeon was available) to reestablish a surgical airway.

**Figure 2:**
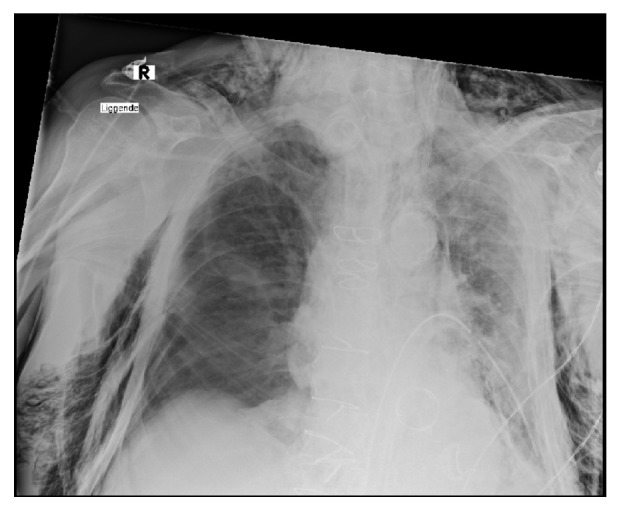
Chest X-ray is showing massive subcutaneous emphysema after CICO and reestablishing a surgical airway.

Meanwhile, the patient became increasingly cardiac rhythm unstable and had a relapse of arterial fibrillation with a frequency of 105 to 200 beats per minute, while holding a mean atrial pressure (MAP) above 65 mmHg with a variable need for inotropic support. Cardiac instability was likely due to subcutaneous emphysema in the mediastinum. FATE (Focus Assessed Transthoracic Echocardiography) was inconclusive as the massive subcutaneous emphysema made it impossible to visualise the heart, as seen on the X-ray Figure 2.

Patient stabilised with increasing MAP and fewer episodes with tachycardia. Therefore, Transesophageal Echocardiography (TEE) was refrained from initially.

## 3. Discussion

The primary surgical tracheostomy was made at the level of the second and third tracheal rings. Incision into trachea was a standard reversed U-incision, and the tracheal tube was inserted without any difficulties or complicating bleeding. Therefore, the local destabilisation of the lumen of the respiratory tract would be expected to be minimal. Nevertheless, a serious complication due to a misplacement of cannula happened. A complication that is generally reported to be fairly low ranged from 0.35 to 2.6% although rates as high as 15% have been reported [4]. The immediate risk to the patient is entirely related to the patient's respiratory status. The addition of subcutaneous emphysema that in its nature presents along with a wide clinical spectrum (e.g., from trivial to lethal) was a severe complicating factor, making the present case life-threatening. In this case, despite the knowledge of potential complications, initial handling relied upon the knowledge of resent unproblematic intubation, and the first attempt to intubation orally was made with a standard laryngoscope. Second intubation attempt, in what proved to be a difficult airway, was made with a video laryngoscope, which evidently increases the glottic view [8] and perhaps the success rate for intubation. Another strategy used was bag/mask ventilation between intubation attempts, as soon as saturation became an issue regardless of the patient not fasting. As suggested in the Vortex tool for airway management the approach of maintaining, convert, and replace was used [9]. At least to some extent, as a laryngeal mask was not introduced at any point. In hindsight, the laryngeal mask would not have solved the increasing subcutaneous emphysema, but it might have bought time to do a proper inspection and to reestablish the surgical tracheostomy [10].

Furthermore, as noted in the case a flexible bronchoscope was requested, and although it was available within the ICU, it did not arrive until measures had been taken to secure the airway. In the situation, the lack of having a flexible bronchoscope ready in hand evident as a direct view of obstruction would have resolved the situation by using video laryngoscope for visualisation of the vocal cords followed by the flexible bronchoscope to clear the obstruction before insertion of the tube. Better yet a tracheal inspection either orally or through the tracheostomy may have resolved the situation before it escalated. As such a minor dose of ketamine would likely have been sufficient to calm the patient allowing the procedure with or without sufficient effect of local anaesthesia.

A Frova or bougie (airway guiding catheter) was never introduced, although it was requested more than once. The reason for this was that the nurses present did not know what it was or where to find it in the difficult airway management cart, even when it was described in detail, which would indicate a need for knowledge of equipment, in particular, the difficult airway trolley. Had it been introduced at an earlier stage, it could potentially have been used instead of the more flexible suction catheter as a guide for the tracheostomy cannula, although the blind procedures, in this case, proved to be unwanted.

This particular case had a successful outcome, but the opposite could have happened. It is likely that the outcome alone is due to the ability of reflection-in-action as the CICO situation occurred. A skill gained through multiple practice scenarios (e.g., simulation) with following reflection-on-action (e.g., debriefing/feedback) [6, 7], which can only be encouraged for all critical events.

## 4. Conclusion

This report highlights the need for preparation and situational awareness. Consequently, this needs to be promoted through deliberate practice and continuing professional development.

We hope that others can learn from this case and will continue to encourage reflective practice. For us, this means to have a flexible bronchoscope as part of plan A when handling problematic tracheostomy airways. Also, due to the rare occurrence of airway crisis and the relatively large turnover of nursing staff, a pictogram (with equipment names) has been attached to the difficult airway trolley for ensuring an accessible overview of the content.

## Additional Points

This case report describes the accidental dislocation of a tracheostomy cannula and airway management in the aftermath of the incident.
